# Response strategies of Ochroma lagopus to short-term low-temperature stress and rewarming: a photosynthesis-antioxidant-carbon network analysis

**DOI:** 10.3389/fpls.2025.1683640

**Published:** 2026-03-30

**Authors:** Yuanxi Liu, Weisong Zhu, Guanben Du, Jialan Chen, Junwen Wu, Rui Shi

**Affiliations:** 1 College of Forestry, Southwest Forestry University, Kunming, Yunnan, China; 2 College of Materials and Chemical Engineering, Southwest Forestry University, Kunming, Yunnan, China; 3 Yunnan Provincial Key Laboratory for Conservation and Utilization of In-forest Resource, Southwest Forestry University, Yunnan Kunming, China

**Keywords:** Ochroma lagopus Swartz, low-temperature stress and rewarming, antioxidant enzyme activity, photosynthesis, non-structural carbohydrates

## Abstract

This study investigated the dynamic responses of photosynthetic physiology, antioxidant system, osmotic regulatory substances, and carbohydrate metabolism in seedlings of the tropical tree species *Ochroma lagopus* Swartz under different low-temperature (3°C, 5°C, 7°C) stress for 24 h and subsequent recovery (12 h, 24 h, 48 h). The results showed that low temperatures significantly reduced chlorophyll a content and the maximum photochemical efficiency of PSII (Fv/Fm). However, carotenoids content increased by 33.97% under 5°C, enhancing photoprotection through the xanthophyll cycle. Photosynthetic parameters recovered rapidly after rewarming from 7°C, while the photosynthetic system collapsed under 3°C due to thylakoid membrane damage. In terms of antioxidant and osmotic regulation: under 3°C stress, superoxide dismutase (SOD) activity increased by 109.70% and proline (PRO) accumulated by 35.66%, alleviating damage by scavenging reactive oxygen species (ROS) and maintaining osmotic pressure. Soluble protein (SP) increased under 5°C, reflecting conservative osmotic regulation. In non-structural carbohydrate (NSCs) metabolism: starch accumulated by 23.08% under 5°C stress, and the soluble sugar/starch ratio increased by 163.99% after recovery, prioritizing energy supply for repair. Under 3°C, NSCs were depleted, blocking repair processes. PCA revealed that the core response dimension during low-temperature stress was “antioxidation - carbon allocation,” which shifted to “photosynthetic repair - energy supply” during recovery. In summary, *O. lagopus* adapts to low temperatures through a “three-level strategy”: efficient repair at 7°C, homeostasis maintenance at 5°C, and stress tolerance at 3°C. This study reveals the precise adaptation mechanism of tropical tree species to low temperatures, providing a theoretical basis for stress resistance management of tropical vegetation.

## Introduction

Temperature is a key environmental factor regulating plant growth, geographical distribution, and productivity (Taylor and Rowley, 1971; Greer et al., 1986). Based on damage mechanisms, low-temperature stress can be divided into chilling injury (above 0°C) and freezing injury (below 0°C). Chilling injury primarily causes reversible damage by disrupting the function of the photosynthetic apparatus (Berry and Björkman, 1980; Zinn et al., 2010), while freezing injury often leads to plant death due to irreversible structural damage caused by intracellular ice formation (Pearce, 2001; Ruelland et al., 2009). A common feature of both is a significant reduction in photosynthetic efficiency, CO_2_ assimilation rate, and photosystem II (PSII) activity (Murata et al., 2007; Ruelland and Zachowski, 2010). Studies have shown that low temperatures can damage all components of the photosynthetic chain, including disruption of the thylakoid electron transport chain, impairment of the carbon reduction cycle, and imbalance in stomatal conductance regulation (Allen and Ort, 2001). The cumulative effect of these damages ultimately leads to photoinhibition in plants (Du et al., 1999). Notably, research on the response mechanisms of the photosynthetic system in tropical plants (especially fast-growing tree species) during the transition phase from chilling to freezing injury (e.g., 5–0°C) remains scarce, and the differences in their specific damage targets compared to temperate plants have not been clarified.

When the damage rate of photosystem II (PSII) exceeds its repair capacity under low-temperature stress, it causes a coordinated decline in photochemical efficiency and photosynthetic capacity, which is a core marker of photoinhibition (Zhou et al., 2013; Kong et al., 2014). Meanwhile, dysfunction of photosystems leads to excessive accumulation of reactive oxygen species (ROS), which trigger peroxidation by attacking membrane lipids (Liu et al., 2019). Malondialdehyde (MDA), as a characteristic product of membrane lipid peroxidation, its content can directly reflect the degree of damage. To resist oxidative damage from ROS, plants have evolved an antioxidant enzyme system centered on superoxide dismutase (SOD), catalase (CAT), and peroxidase (POD) (Duan et al., 2012; Noman et al., 2017). The dynamic balance between ROS production and scavenging is crucial for maintaining the stability of the photosynthetic system. When stress intensity breaks this balance (e.g., scavenging capacity decreases due to inhibited enzyme activity), it triggers photooxidation, ultimately leading to irreversible inactivation of the photosynthetic system, with the degradation of photosystem I (PSI) being particularly significant. However, existing studies have mostly focused on changes in the activity of a single antioxidant enzyme, and systematic analysis of the coupled regulatory mechanisms between the antioxidant system and photosystem repair under low-temperature stress is still lacking. In addition, osmotic adjustment under low-temperature stress is an important part of plant adaptation strategies. Soluble proteins (SP) and proline (PRO) maintain the structural integrity of cell membranes and protoplasts by coordinately regulating cell osmotic pressure, reducing the freezing point of cytoplasm (Liu et al., 2019). Among them, soluble protein content is positively correlated with the expression levels of antifreeze proteins (AFPs) and cold shock proteins (CSPs), and can be used as a key indicator to evaluate the low-temperature regulation ability of plants (Lu et al., 2024; Jones and Mullet, 1995). However, for tropical tree species, the metabolic flux allocation priority between osmotic regulatory substances (such as proline) and antioxidant enzyme systems under low-temperature stress, as well as their linkage mechanism with carbon metabolism, have not been clarified, which limits the comprehensive understanding of their low-temperature adaptation strategies.

Carbon products fixed by trees through photosynthesis can be divided into structural carbohydrates (SCs) and nonstructural carbohydrates (NSCs) based on their functions and forms (Martínez-Vilalta et al., 2016). Among them, NSCs, as the core energy pool for plant growth and metabolism, mainly consist of soluble sugars (responsible for transport and immediate utilization) and starch (undertaking long-term storage functions) (Furze et al., 2019). Therefore, analyzing the dynamic changes in NSCs concentration in plant tissues can not only reveal the carbon balance mechanism but also clarify the physiological and ecological response strategies of plants under environmental stress. As the main organ for photosynthesis (Liu et al., 2017), leaf NSCs and their components are both direct manifestations of photosynthetic products and carbon skeletons for synthesizing various biomolecules. Their content fluctuations can sensitively reflect the adaptive adjustments of plants to changes in the external environment (Ramírez-Briones et al., 2017). Especially under stress conditions, the reserve level and conversion efficiency of leaf NSCs are crucial for determining plant viability, but their regulatory mechanisms in tropical fast-growing tree species remain unclear.

Existing studies have shown that environmental stress significantly reshapes the allocation pattern of NSCs. During the dry season, most tree species maintain basic metabolism by mobilizing stored NSCs, leading to a decrease in their total amount (Sala et al., 2010; Liu Y. et al., 2023). This imbalance in carbon consumption ultimately increases tree mortality (McDowell et al., 2008; Oleksyn et al., 2000). In low-temperature environments, the needle starch content of Scots pine (Pinus sylvestris) begins to accumulate when the daily minimum temperature remains above 0°C, peaks before new leaf germination (with the highest accumulation in one-year-old needles), and gradually decreases to a minimum in late spring and early summer due to growth consumption. This dynamics reflects the active adaptation strategy of temperate tree species to seasonal low temperatures. The shading experiment by Liu et al. (2024) showed that *Pinus yunnanensis* seedlings adapt to light gradient changes by increasing the proportion of NSCs allocated to morphogenesis and accelerating the conversion of soluble sugars to starch. However, under 95% shading, carbon budget imbalance due to insufficient photosynthetic carbon input ultimately inhibits growth. These studies suggest that the metabolic plasticity of NSCs is an important basis for plants to adapt to stress. However, systematic research on the NSCs allocation strategy of tropical tree species under low-temperature stress (e.g., whether starch accumulation is the result of active regulation or passive damage) and its coupling mechanism with photosynthetic efficiency is still lacking.

*O. lagopus*, a typical fast-growing tropical arbor, is native to tropical rainforests in Central and South America, and has been successfully introduced and cultivated in tropical and subtropical regions such as Yunnan and Hainan in China. This species has strict habitat adaptability: the optimal growth temperature is 25–30°C, the annual precipitation requirement is ≥1500 mm, and it is significantly sensitive to low temperatures—growth stagnates below 10°C, which is closely related to its evolutionary background of tropical origin. As a pioneer species in niche competition, *O. lagopus* quickly occupies forest gap spaces with an amazing growth rate of 10–15 cm in annual DBH (diameter at breast height). Its xylem density is only 0.1–0.2 g·cm^−3^, making it one of the lightest commercial woods, with irreplaceable economic value in aerospace materials, buffer packaging, and other fields (Liu CA et al., 2024). Current research on *O. lagopus* mainly focuses on specific physiological processes and environmental responses. Liu et al. (2024) explored the ecological regulatory mechanisms during seed germination, Liu et al. (2025a) analyzed the physiological adaptation strategies under repeated drought stress, Wen et al. (2025) and Chen et al. (2025) studied the effects of nitrogen addition on its growth, Samuel and Alejandro (2024) focused on its survival dynamics, and Niloufar et al. (2022) focused on the improvement of wood physical properties. However, systematic research on low-temperature stress remains blank, especially the damage mechanism of natural extreme low-temperature events on *O. lagopus* has not been clarified. Xishuangbanna, Yunnan, the main cultivation area of *O. lagopus* in China, has experienced frequent extreme low-temperature events in recent winter and spring, with the recorded minimum temperature reaching 3.5°C (https://m.yunnan.cn/system/2024/03/26/032988660.shtml). Such low temperatures have caused significant yield reductions in local tropical crops (e.g., tea trees, rubber trees) (https://china.huanqiu.com/article/9CaKrnJTtNv). In production practice, it has been observed that after *O. lagopus* is exposed to temperatures below 5°C for 48 hours, the shoot tips show irreversible wilting—even if the temperature rises, growth cannot be restored. This phenomenon suggests that there is a critical threshold in its low-temperature response, but the specific damage nodes and recovery mechanisms have not been clarified. Notably, *O. lagopus* lacks the cold acclimation ability unique to temperate plants, and its low-temperature response mode can reflect the “primitive strategy” of tropical species in coping with low-temperature stress, providing key comparative materials for analyzing the evolutionary differentiation of cold resistance mechanisms among tree species in different climate zones. Based on the above production needs and theoretical gaps, this study designed a simulation experiment involving 24-hour gradient low-temperature stress and 48-hour recovery. The core objectives are: (1) to clarify the self-recovery potential of *O. lagopus* after short-term low-temperature stress; (2) to quantify the recovery dynamics under different temperature gradients and their key physiological thresholds; (3) to reveal the irreversible critical point of low-temperature damage in tropical fast-growing tree species. This research can not only provide technical support for low-temperature early warning in *O. lagopus* cultivation but also fill the research gap in the low-temperature adaptation mechanism of tropical tree species.

## Materials and methods

### Study area, plant materials, and experimental design

The experiment was conducted on the campus of Southwest Forestry University in Kunming, Yunnan Province (E102°46′, N25°03′), at an altitude of 1964 m, with an annual average temperature of 16.5°C and annual precipitation of 1035 mm. Two-month-old *O. lagopus* seedlings, propagated from seeds and cultivated at the Xishuangbanna Tropical Botanical Garden, Chinese Academy of Sciences, were transported to the Arboretum of Southwest Forestry University on July 30, 2024, for transplanting. The planting soil was a mixture of red soil and humus in a 3:2 ratio, with total carbon content of 3.26 g·kg^−1^, total nitrogen of 5.98 g·kg^−1^, total phosphorus of 0.62 g·kg^−1^, and pH 7.65. On September 29, 2024, uniformly sized and vigorously growing seedlings (with an average height of 22.1 cm and basal diameter of 8.25 mm) were transferred to the Key Laboratory of National Forestry and Grassland Administration for Biodiversity Conservation in Southwest China, College of Forestry, Southwest Forestry University, for low-temperature stress experiments.

Four temperature gradients were set: CK (25°C, control), T7 (7°C), T5 (5°C), and T3 (3°C). Seedlings were acclimated in artificial climate chambers (produced by Wuhan Ruihua Instrument Co., Ltd.) at 25°C/15°C (day/night) for 5 days prior to the experiment.

Low-temperature stress and recovery treatments: The stress experiment was initiated at 8:00 on October 1, 2024, in artificial climate chambers set to 25°C, 7°C, 5°C, and 3°C. Five *O. lagopus* seedlings were randomly assigned to each chamber, with the following conditions: light intensity of 10000 lx, relative humidity of 50%, and a photoperiod of 16 h light/8 h dark. After 24 h of low-temperature stress (L24h), seedlings were allowed to recover at 25°C, and leaf physiological indices were measured at 12 h (R12h), 24 h (R24h), and 48 h (R48h) of recovery.

### Determination of leaf physiological and biochemical indices

At the end of each treatment, chlorophyll fluorescence parameters (initial fluorescence [Fo], maximum fluorescence (Fm), variable fluorescence (Fv) were measured using a FluorCam chlorophyll fluorometer (PSI, Czech Republic). The potential photochemical activity of PSII (Fv/Fo) and the maximum photochemical efficiency of PSII (Fv/Fm) were calculated (Goltsev et al., 2016). For enzyme activity assays, fresh apical leaves from each treatment were sampled, wrapped in tin foil, and stored in liquid nitrogen. Other leaves were oven-dried at 120°C for 30 min to inactivate enzymes, then dried to constant weight at 80°C; the dried samples were ground and sieved for nonstructural carbohydrate (NSCs) analysis.

After the experiment, fresh leaves from each treatment were cleaned, and 0.5 g of leaf tissue was weighed and chopped in a mortar. Chlorophyll a, chlorophyll b, total chlorophyll (a+b), and carotenoids contents were determined by ethanol extraction, with absorbance measured at 665 nm, 649 nm, and 470 nm using a UV-visible spectrophotometer (Smillie and Hetherington, 1983).

Superoxide dismutase (SOD) activity was measured using the nitroblue tetrazolium (NBT) method (Lei et al., 2006). Peroxidase (POD) activity was determined via the guaiacol method (Sancho et al., 1996). Malondialdehyde (MDA) content was assayed using the thiobarbituric acid (TBA) method (Lei et al., 2006). Proline (PRO) content was measured by the ninhydrin method (Bates et al., 1973). Soluble protein (SP, mg·g^−1^ FW) content was determined according to Lei et al. (2006). Catalase (CAT) activity was analyzed using the potassium permanganate method (Lei et al., 2006).

### Statistical analysis

Before subsequent statistical analyses, the Kolmogorov-Smirnov test was used to assess data normality and homogeneity of variances. Multiple comparisons of mean values were performed using Duncan’s test. Principal component analysis (PCA) of all leaf indices in *O. lagopus* was conducted using the R programming language. All statistical analyses were performed using SPSS 20.0 software (IBM SPSS Statistics, USA), with statistical significance set at *p<*0.05. Data are presented as “mean ± standard deviation,” and bar graphs were generated using GraphPad Prism 8 software.

## Results

### Effects of low-temperature stress and recovery time on leaf chlorophyll content in *O. lagopus* seedlings

**Figure 1:**
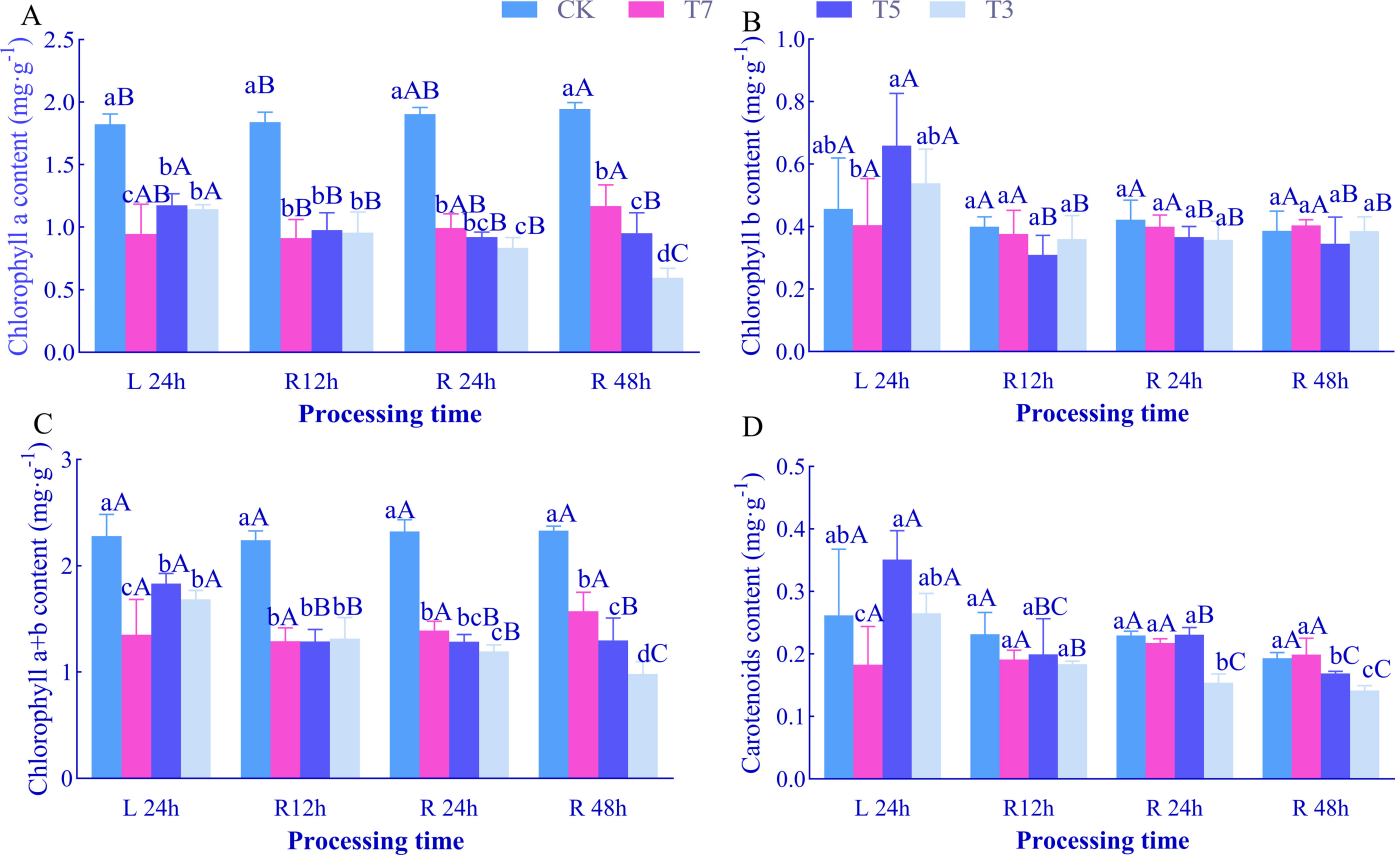
Effects of low-temperature stress and recovery time on chlorophyll content in leaves of *O. lagopus* seedlings. **(A)** Chlorophyll a; **(B)** Chlorophyll b; **(C)** Chlorophyll a+b; **(D)** Carotenoids. The error bars indicate the standard deviation of the mean (n = 5). Different lowercase letters (a, b, c…) indicate significant differences among different temperature gradients at the same time point (*p<*0.05), while different uppercase letters (A, B, C…) denote significant differences among different durations at the same temperature (*p<*0.05).

After 24 h of low-temperature stress (L24h), chlorophyll a and total chlorophyll a+b contents in *O. lagopus* seedling leaves showed a trend of first decreasing and then increasing with decreasing temperature (**Figures 1A, C**). Compared to the control (CK), these contents were significantly reduced under T7, T5, and T3 treatments by 48.05%, 35.51%, 37.22% (for chlorophyll a) and 40.69%, 19.50%, 26.14% (for total chlorophyll), respectively (*p<*0.05). Chlorophyll b and carotenoids contents exhibited a trend of first decreasing, then increasing, and then decreasing again (**Figures 1B, D**): they reached minimum values under T7, with significant reductions of 11.25% (chlorophyll b) and 30.26% (carotenoids) compared to CK (*p<*0.05), and peaked under T5, with significant increases of 44.55% (chlorophyll b) and 33.97% (carotenoids) relative to CK. At each recovery time point, chlorophyll a and total chlorophyll a+b contents decreased significantly with decreasing temperature (*p<*0.05). Chlorophyll b content showed no significant difference from CK at R12h, R24h, and R48h during the recovery phase (**Figures 1A, C**). Carotenoids content showed no significant difference among treatments at R12h; at R24h, it was significantly reduced by 32.77% under T3; at R48h, it was significantly decreased by 12.49% (T5) and 29.74% (T3) compared to CK (**Figures 1B, D**).

With increasing recovery time, under T5, chlorophyll a content decreased significantly at R12h and R48h (*p<*0.05). Under T3, chlorophyll a content decreased significantly at R12h, R24h, and R48h (*p<*0.05), reaching a minimum of 0.60 mg·g^−1^ at R48h, which was 16.41%, 27.03%, and 47.95% lower than that at L24h, respectively. Total chlorophyll (a+b) and carotenoids contents showed no significant difference under T7 with increasing recovery time, but decreased significantly under T5 and T3 (*p<*0.05). Compared to L24h, total chlorophyll (a+b) decreased by 53.05% (T5) and 33.18% (T3), the chlorophyll a+b and carotenoids content reached its minimum value after R24h.

### Effects of low-temperature stress and recovery time on leaf fluorescence characteristics in *O. lagopus* seedlings

**Figure 2:**
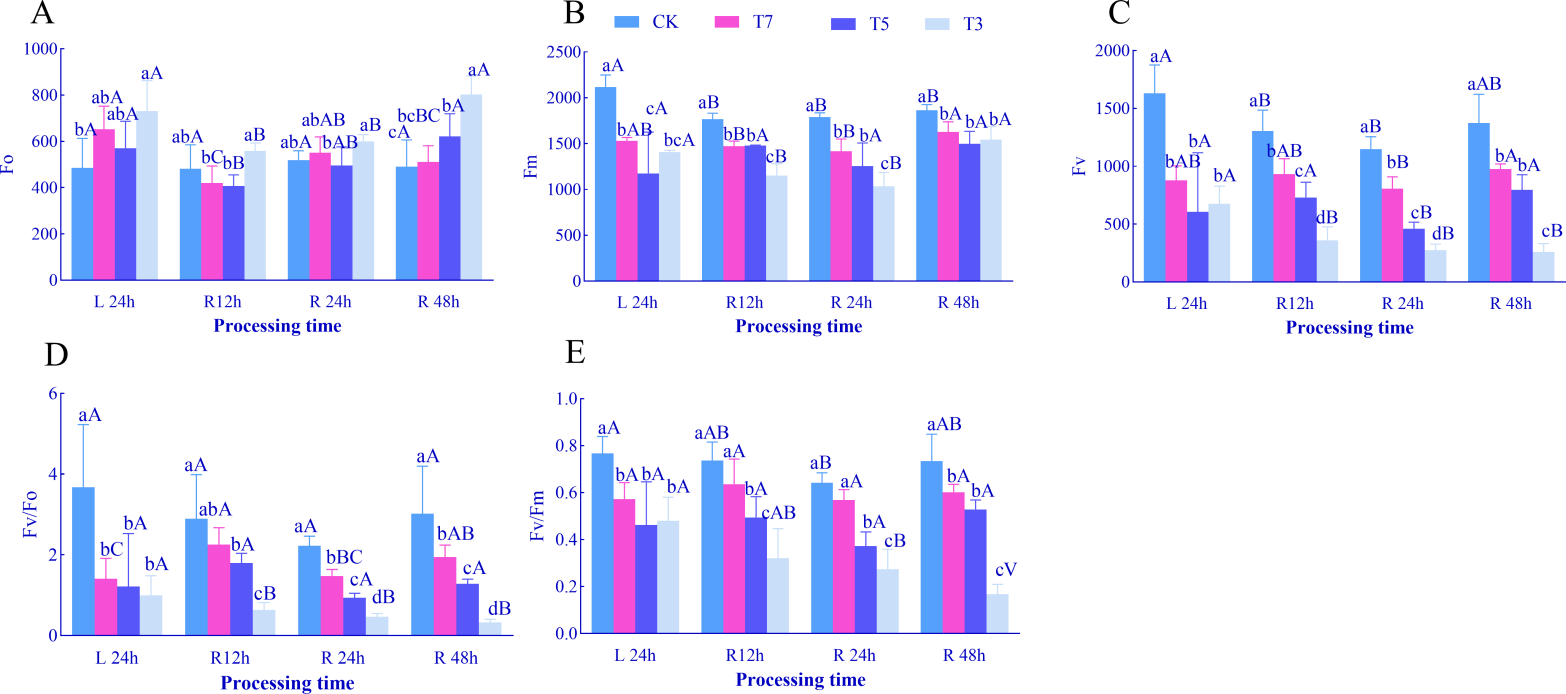
Effects of low-temperature stress and recovery time on the fluorescence characteristics of *O. lagopus* seedling leaves. **(A)** Fo; **(B)** Fm; **(C)** Fv; **(D)** Fv/Fo; **(E)** :Fv/Fvm. The error bars indicate the standard deviation of the mean (n = 5). Different lowercase letters (a, b, c…) indicate significant differences among different temperature gradients at the same time point (*p<*0.05), while different uppercase letters (A, B, C…) denote significant differences among different durations at the same temperature (*p<*0.05).

After 24 h of low-temperature stress (L24h), with decreasing temperature, the initial fluorescence (Fo) of *O. lagopus* seedling leaves showed a trend of first increasing, then decreasing, and then increasing again. Under T3 treatment, Fo was significantly higher than that of CK by 50.32% (**Figure 2A**). Maximum fluorescence (Fm), variable fluorescence (Fv), and the maximum photochemical efficiency of PSII (Fv/Fm) exhibited a trend of first decreasing and then increasing, all remaining lower than those in the CK group. They reached the minimum values under T5 treatment, which were significantly lower than CK by 44.38%, 62.80%, and 39.77%, respectively (**Figures 2B–D**). The potential photochemical activity of PSII (Fv/Fo) showed a continuous decreasing trend, with a significant reduction of 72.88% under T3 compared to CK (**Figure 2E**). At each recovery time point, with decreasing temperature, Fm, Fv, Fv/Fm, and Fv/Fo showed a continuous decreasing trend, while Fo fluctuated (increasing and decreasing alternately).

With increasing recovery time: Under T7 treatment, Fm, Fv, and Fv/Fm showed no significant differences. Fo decreased significantly by 35.76% and 21.64% at R12h and R48h, respectively, while Fv/Fo increased significantly by 60.38% and 37.94% at R12h and R48h (*p<*0.05). Under T5 treatment, Fm, Fv/Fo, and Fv/Fm showed no significant differences. Fo decreased significantly after R12h, and Fv decreased significantly after R24h (*p<*0.05). Under T3 treatment, Fo and Fm decreased significantly after R12h and R48h; Fv and Fv/Fo decreased significantly at R12h, R24h, and R48h; Fv/Fm decreased significantly after R24h and R48h (*p<*0.05).

### Effects of low-temperature stress and recovery time on leaf CAT, SOD, POD activities, and MDA content in *O. lagopus* seedlings

**Figure 3:**
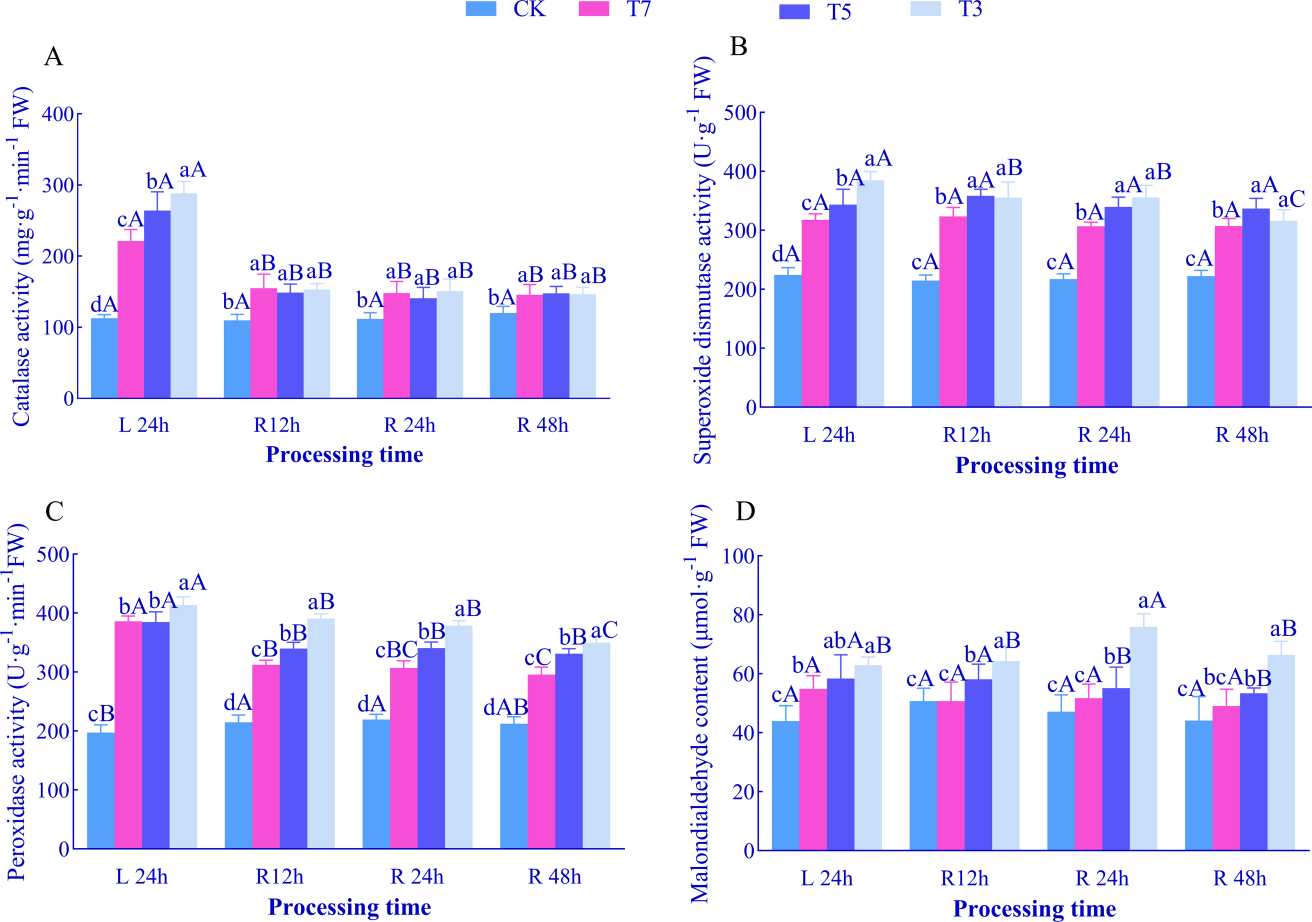
Effects of low-temperature stress and recovery time on CAT, SOD, POD, and MDA in the leaves of *O. lagopus* seedlings. **(A)** CAT; **(B)** SOD; **(C)** POD; **(D)** MDA. The error bars indicate the standard deviation of the mean (n = 5). Different lowercase letters (a, b, c…) indicate significant differences among different temperature gradients at the same time point (*p<*0.05), while different uppercase letters (A, B, C…) denote significant differences among different durations at the same temperature (*p<*0.05).

After 24 h of low-temperature stress (L24h), with decreasing temperature, the activities of CAT, SOD, POD, and MDA content in *O. lagopus* seedling leaves showed a continuous increasing trend (**Figure 3**). They reached maximum values under T3 treatment, which were significantly higher than those in the CK group by 71.57% (CAT), 109.70% (SOD), 35.66% (POD), and 5.49% (MDA), respectively (*p<*0.05). At each recovery time point, with decreasing temperature, CAT, SOD, POD activities, and MDA content all increased significantly, with the highest values consistently observed under T3 treatment.

With increasing recovery time, compared to L24h: Under T7 treatment, CAT and POD activities decreased significantly at R12h, R24h, and R48h (*p<*0.05), reaching minimum values at R48h, which were 34.13% and 23.40% lower than those at L24h, respectively. SOD activity and MDA content showed no significant differences across all recovery time points. Under T5 treatment, CAT and POD activities decreased significantly at R12h, R24h, and R48h (*p<*0.05). MDA content decreased significantly by 5.57% and 8.59% at R24h and R48h, respectively, while SOD activity showed no significant differences across all recovery time points. Under T3 treatment, CAT, SOD, and POD activities decreased significantly at R12h, R24h, and R48h (*p<*0.05). MDA content showed no significant differences at R12h and R48h but increased significantly by 20.67% at R24h.

### Effects of low-temperature stress and recovery time on leaf 0smotic regulation in *O. lagopus* seedlings

**Figure 4:**
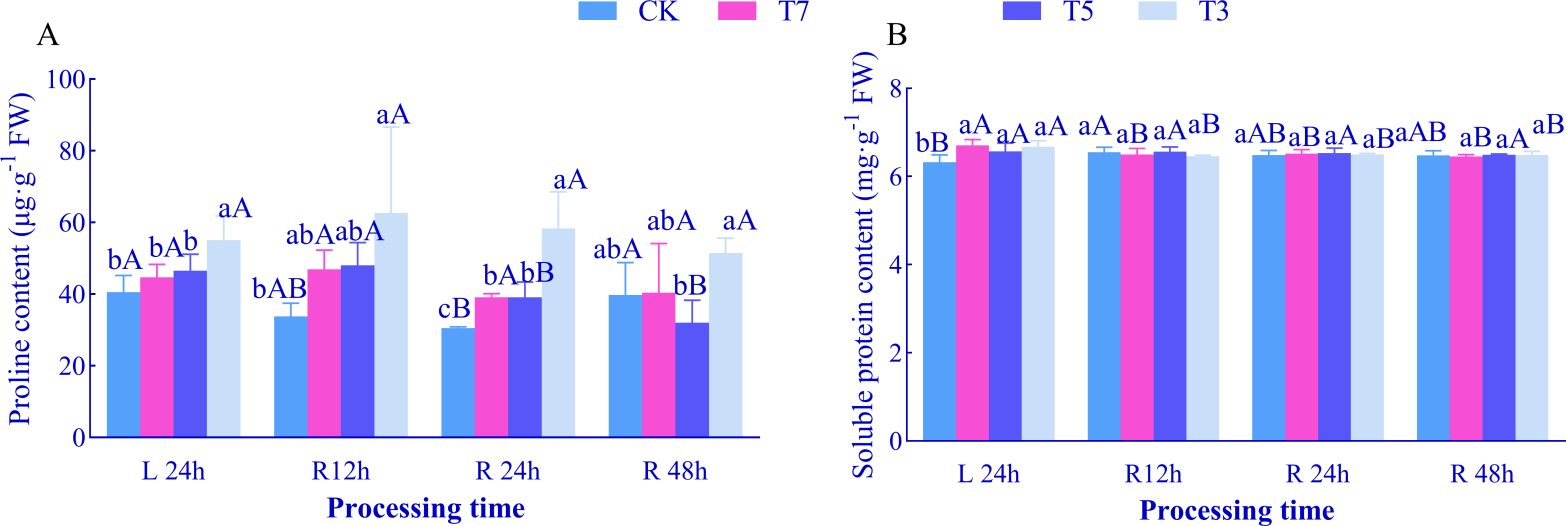
Effect of low temperature stress and recovery time on Pro and SP content in leaves of *O. lagopus* seedlings. **(A)** PRO; **(B)** SP. The error bars indicate the standard deviation of the mean (n = 5). Different lowercase letters (a, b, c…) indicate significant differences among different temperature gradients at the same time point (*p<*0.05), while different uppercase letters (A, B, C…) denote significant differences among different durations at the same temperature (*p<*0.05).

After 24 h of low-temperature stress (L24h), with decreasing temperature, the PRO content in *O. lagopus* seedling leaves showed a continuous increasing trend, reaching a maximum of 55.04 mg·g^−1^ under T3 treatment, which was significantly higher than that in the CK group by 35.66% (*p<*0.05). However, there was no significant difference in PRO content between T7, T5 treatments and CK (**Figure 4A**). The SP content exhibited a trend of first increasing, then decreasing, and then increasing again. Compared to CK, SP content increased significantly under T7, T5, and T3 treatments (*p<*0.05) (**Figure 4B**). At each recovery time point, with decreasing temperature: At R12h and R24h, PRO content increased significantly, peaking under T3 treatment (62.64 mg·g^−1^ and 58.25 mg·g^−1^, respectively), which were 85.31% and 90.99% higher than those in CK (*p<*0.05). At R48h, PRO content in T5 treatment decreased significantly by 19.42% compared to CK. SP content showed no significant differences across all recovery time points.

With increasing recovery time, compared to L24h: In CK treatment, PRO content decreased significantly by 24.82% at R24h, while SP content increased significantly after R12h. In T7 treatment, SP content decreased significantly at all recovery time points, reaching a minimum of 6.45 mg·g^−1^ at R48h, which was 3.71% lower than that at L24h (*p<*0.05), while PRO content showed no significant differences across all recovery time points. In T5 treatment, PRO content decreased significantly by 15.87% and 31.09% at R24h and R48h, respectively (*p<*0.05), while SP content showed no significant differences across all recovery time points. In T3 treatment, SP content decreased significantly at all recovery time points, while PRO content showed no significant differences across all recovery time points.

### Effects of low-temperature stress and recovery time on leaf NSCs content in *O. lagopus* seedlings

**Figure 5:**
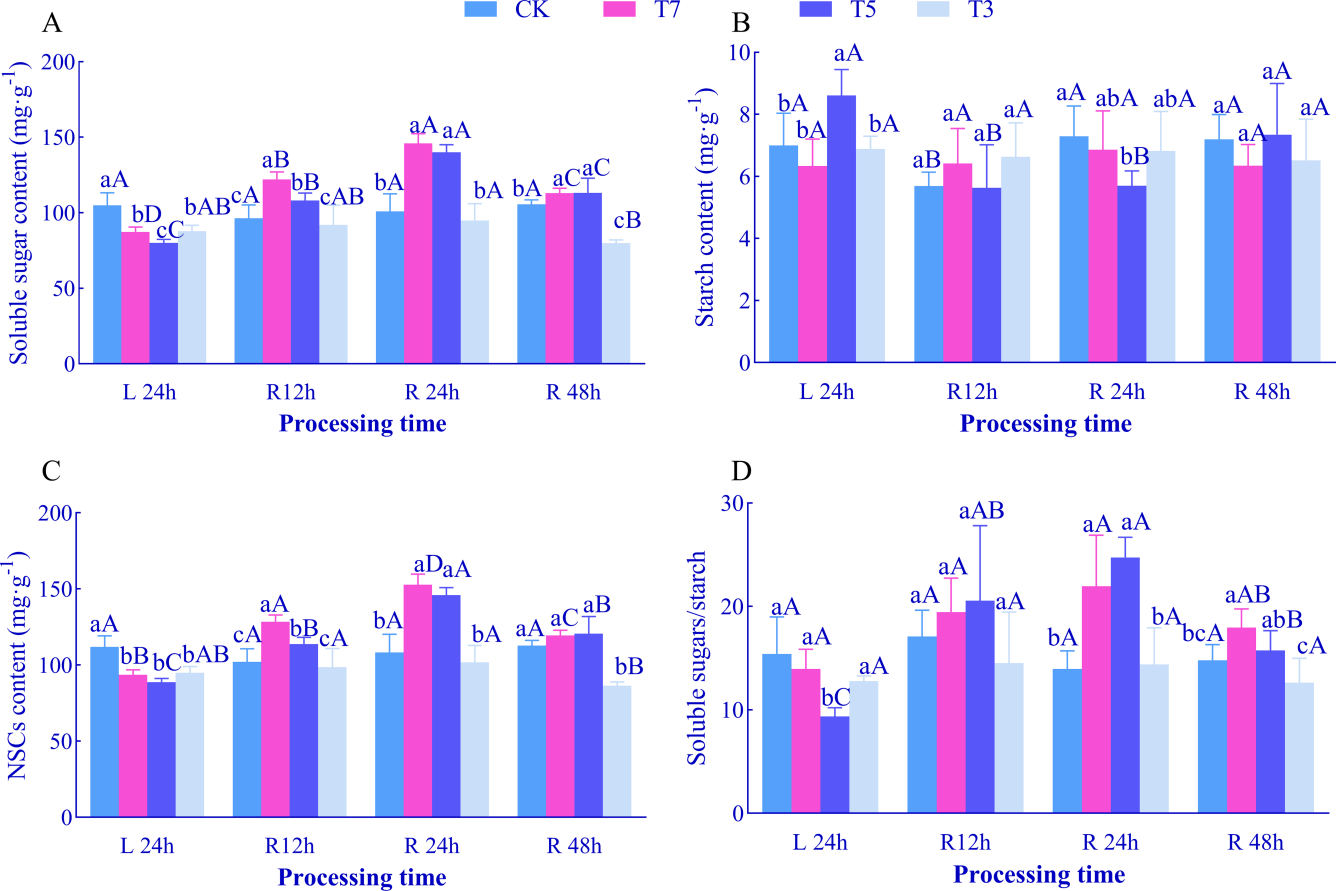
Effects of low-temperature stress and recovery time on NSC content in leaves of *O. lagopus* seedlings. **(A)** Soluble sugars; **(B)** Starch; **(C)** NSCs; **(D)** Soluble sugars/starch. The error bars indicate the standard deviation of the mean (n = 5). Different lowercase letters (a, b, c…) indicate significant differences among different temperature gradients at the same time point (*p<*0.05), while different uppercase letters (A, B, C…) denote significant differences among different durations at the same temperature (*p<*0.05).

After 24 h of low-temperature stress (L24h), with decreasing temperature, the contents of soluble sugars and total NSCs in *O. lagopus* seedling leaves showed a trend of first decreasing and then increasing (**Figures 5A, C**). Compared to CK, both were significantly reduced under T7, T5, and T3 treatments (*p<*0.05). Under T5 treatment, starch content increased significantly by 23.08% (**Figure 5B**), while the soluble sugar/starch ratio decreased significantly by 39.26% (**Figure 5D**); no significant differences were observed between T7, T3 treatments and CK for these indices. At each recovery time point, with decreasing temperature: Soluble sugar content, total NSCs content, and the soluble sugar/starch ratio showed a trend of first increasing and then decreasing, with significant increases under T7 and T5 treatments (except for NSCs content after R48h). Starch content fluctuated (increasing and decreasing alternately) across recovery time points; under T5 treatment, it decreased significantly after R24h.

With increasing recovery time, compared to L24h: Under T7 treatment, soluble sugar and total NSCs contents increased significantly at all recovery time points, peaking at R24h (6.55 mg·g^−1^ and 152.71 mg·g^−1^, respectively), which were 90.94% and 63.29% higher than those at L24h. Starch content and the soluble sugar/starch ratio showed no significant differences across recovery time points. Under T5 treatment, soluble sugar content, total NSCs content, and the soluble sugar/starch ratio increased significantly, all peaking at R24h, with increases of 74.91%, 64.36%, and 163.99% compared to L24h, respectively. Starch content decreased significantly by 34.53% and 33.86% at R12h and R24h, respectively. Under T3 treatment, soluble sugar and total NSCs contents reached their minimum values at R48h, with no significant differences observed at other recovery stages.

### Principal component analysis of leaf indices in *O. lagopus* seedlings under low-temperature stress and recovery

**Figure 6:**
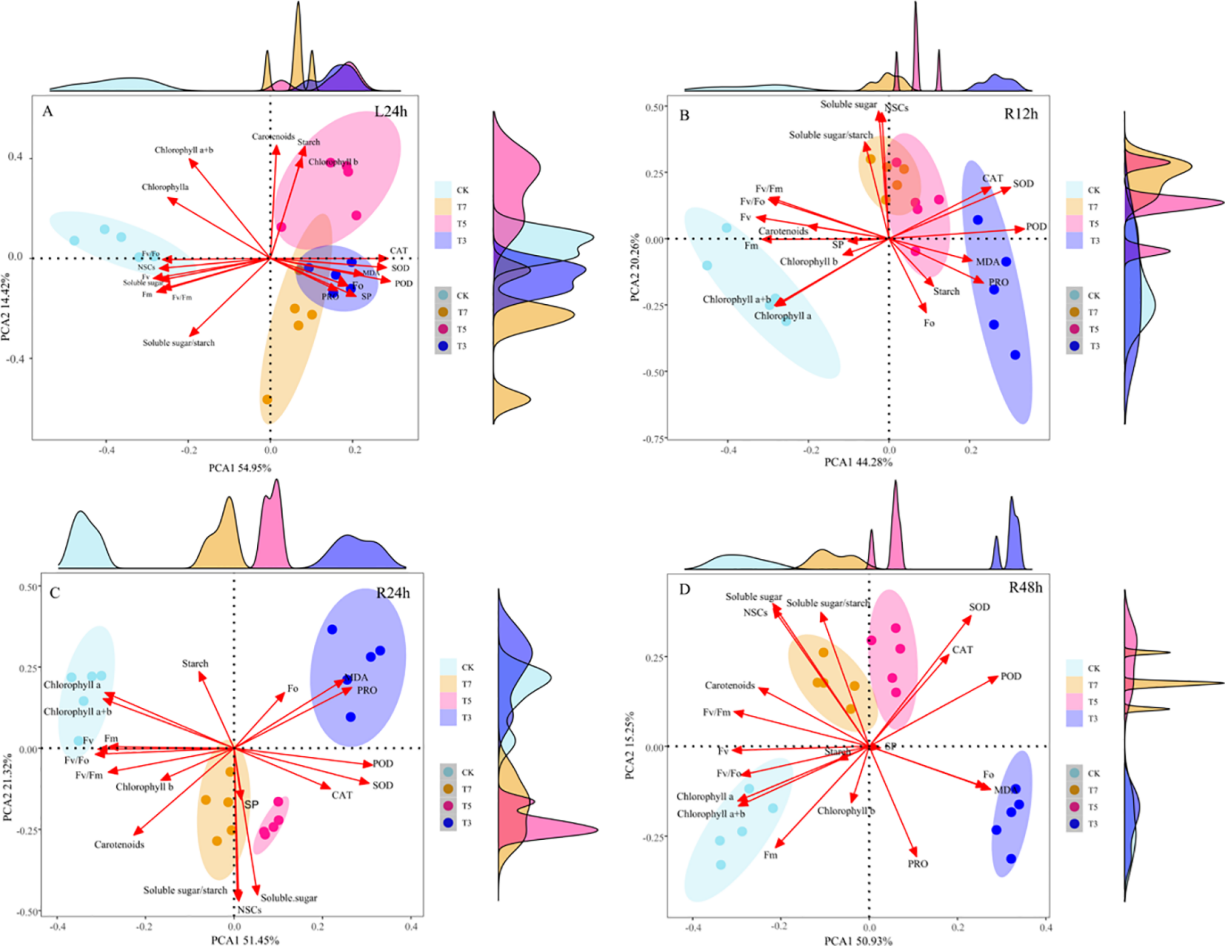
Principal component analysis of various indicators of *O. lagopus* seedlings’ leaves under low-temperature stress and recovery time. **(A)** L24h; **(B)** R12h; **(C)** R24h; **(D)** R48h.

Principal component analysis (PCA) of leaf indices in *O. lagopus* seedlings under low-temperature stress and recovery is shown in **Figure 6**. The cumulative variance contribution rates of the first two principal components (PC1 and PC2) at L24h, R12h, R24h, and R48h were 69.37%, 64.54%, 72.77%, and 66.18%, respectively, indicating that these two components effectively explain the response characteristics of *O. lagopus* seedlings to low-temperature stress and recovery time. At L24h (**Figure 6A**), PC1 was dominated by indices with high weight coefficients: POD, SOD, CAT, soluble sugars, NSCs, and variable fluorescence (Fv). PC2 was primarily associated with the soluble sugar/starch ratio, carotenoids, total chlorophyll (a+b), and starch. At R12h (**Figure 6B**), PC1 had high weight coefficients for POD, SOD, Fv, and maximum fluorescence (Fm). PC2 was dominated by soluble sugars and NSCs. At R24h (**Figure 6C**), PC1 was strongly associated with POD, SOD, Fv, the potential photochemical activity of PSII (Fv/Fo), and chlorophyll a. PC2 was primarily driven by NSCs, soluble sugars, and the soluble sugar/starch ratio. At R48h (**Figure 6D**), PC1 had high weight coefficients for POD, MDA, Fv, and chlorophyll a. PC2 was dominated by PRO, NSCs, and soluble sugars.

## Discussion

### Effects of low-temperature stress and recovery duration on photosynthetic pigments and chlorophyll fluorescence parameters in *O. lagopus* seedlings

Chlorophyll a and chlorophyll b are core pigments for light energy capture, and their contents directly determine the efficiency of light absorption and conversion by photosystems (Croft et al., 2017). In addition to assisting in capturing blue-violet light, carotenoids quench excess excitation energy and scavenge ROS through the xanthophyll cycle, serving as key executors of photoprotective mechanisms (Ledford and Niyogi, 2005). In this study, after 24 h of low-temperature stress, chlorophyll a and total chlorophyll contents decreased significantly under T7 (7°C) and T3 (3°C) treatments, while the chlorophyll b content in the T5 (5°C) treatment group exhibited a unique pattern of initial decrease followed by increase (**Figures 1A, C**). This result is partially consistent with the general conclusion that “low temperatures hinder chlorophyll synthesis by inhibiting protochlorophyllide oxidoreductase activity” (Brunetti et al., 2019). However, the observed recovery in T5 indicates that mild low temperatures may activate a compensatory mechanism for chlorophyll synthesis. We hypothesize that the upregulation of glutamyl-tRNA reductase expression under 5°C partially offsets the inhibitory effect of low temperatures on pigment synthesis, whereas the 3°C treatment exacerbates chlorophyll degradation due to thylakoid membrane disintegration (Tang et al., 2021). Carotenoids content decreased by 30.26% under T7 but increased by 33.97% under T5, and recovered to CK levels under T3 treatment, showing a “low-temperature intensity-dependent fluctuation” (**Figure 1D**). This dynamics may be related to the activation threshold of the xanthophyll cycle: carotenoids accumulation under T5 may enhance non-photochemical quenching (NPQ) efficiency by promoting zeaxanthin synthesis (Liu et al., 2001), while the decrease observed in T3 treatment compared to T5 reflects dual stressors—reduced *de novo* synthesis due to inhibited synthase activity and accelerated carotenoids consumption by persistent ROS outbursts (Xiang et al., 2023; Li et al., 2025). Changes in chlorophyll fluorescence parameters further revealed the functional status of PSII: a decrease in Fv/Fm (maximum photochemical efficiency of PSII) indicates damage to reaction center structures or blockage of the electron transport chain (Heber et al., 2001), while a decline in Fv/Fo (potential photochemical activity of PSII) suggests impaired efficiency of light energy transfer from antenna pigments to reaction centers (Björkman and Demmig, 1987). The coordinated decrease in Fv/Fm and Fv/Fo under T5 and T3 (**Figures 2D, E**) confirms that the light energy conversion and electron transport capacities of PSII decline synchronously under low-temperature stress, with a more significant reduction under 3°C, indicating a dose effect of extreme low temperatures on photosystem damage.

The dynamic differences during the recovery phase further highlight the critical role of temperature thresholds: under T7 treatment, chlorophyll b and carotenoids rapidly recovered to control levels (**Figures 1B, D**), accompanied by a decrease in Fo and an increase in Fv/Fo (**Figures 2A, D**), indicating that the photosystem damage caused by 7°C stress is reversible, similar to the cold adaptation repair mechanism of temperate plants (Yan et al., 2021).Under T5, photosynthetic pigments and fluorescence parameters remained stable during recovery but did not reach control levels, suggesting that 5°C stress induces a “conservative repair” strategy—reducing ROS production by downregulating photochemical reaction rates (Chaves et al., 2003). This mechanism, which sacrifices short-term photosynthetic efficiency to maintain structural integrity, may represent an adaptive strategy of tropical tree species in response to occasional low temperatures (Zhang et al., 2024). In contrast, under T3, chlorophyll a continued to decrease to 0.60 mg·g^−1^ (**Figure 1A**), carotenoids to 0.22 mg·g^−1^ (**Figure 1D**), and Fv/Fm and Fv/Fo declined persistently, revealing irreversible damage caused by 3°C stress: peroxidation of thylakoid membrane lipids led to the disintegration of chlorophyll-protein complexes, creating a vicious cycle of photochemical system collapse and loss of photoprotective capacity, ultimately inhibiting the production of photosynthetic “assimilatory power” (ATP and NADPH) (Wang et al., 2003).

### Effects of low-temperature stress and recovery time on leaf physiological and biochemical properties of *O. lagopus* seedlings

Non-structural carbohydrates (NSCs), as core substances in plant energy metabolism, their dynamic balance between synthesis and consumption directly regulates growth and development processes (Liu YX et al., 2024; Liu et al., 2023). When abiotic stress intensity exceeds the plant’s regulatory threshold, the metabolic homeostasis of NSCs is disrupted (Liu et al., 2025b); persistent depletion can lead to carbon starvation, ultimately resulting in plant death (McDowell, 2011). In this study, soluble sugar and NSCs content showed a decreasing trend under T7 treatment compared to the control group, while starch content and the soluble sugar/starch ratio remained unchanged, indicating that mild low temperatures have limited inhibitory effects on carbon assimilation. Under T5 conditions, soluble sugars, NSCs, and the soluble sugar/starch ratio continued to decrease, while starch content increased. This indicates that moderate low temperatures promote the conversion of carbon assimilation products into starch storage, with carbon allocation prioritizing energy reserves over immediate consumption. This strategy is similar to the starch accumulation pattern in temperate plants during early cold acclimation (Xiong et al., 2002). However, as a tropical tree species, *O. lagopus* starch synthesis may not rely on low-temperature-induced gene expression (e.g., upregulation of GBSS) but rather on homeostatic regulation of metabolic enzyme activity (Zhang et al., 2024). Under T3, soluble sugars and NSCs significantly decreased while starch content remained stable, reflecting impaired photosynthetic carbon fixation and intensified respiratory consumption under severe low temperatures, and suggesting disruption of carbon metabolic pathways due to chloroplast structural damage (Chaves et al., 2003). The restructuring of carbon metabolism during the rewarming phase further reveals the temperature dependence of adaptive strategies: under T7 treatment, soluble sugars peaked at R24h, and NSCs increased synchronously, while starch content remained stable (**Figure 5**), indicating that photosynthetic carbon assimilation rapidly recovered after mild low temperatures, with carbon flow primarily directed toward soluble sugars. This provides energy substrates for PSII repair (such as D1 protein synthesis) and forms a metabolic coupling with the rapid recovery of chlorophyll b (**Figure 1B**).Under T5, soluble sugars and NSCs continued to increase while starch slightly decreased (**Figure 5**), suggesting starch degradation into soluble sugars via β-amylase catalysis to supply energy for photoprotective mechanisms (e.g., xanthophyll cycle), reflecting a shift in carbon allocation from “storage during stress” to “consumption during recovery”—consistent with the “post-stress carbon metabolic reconfiguration” mechanism proposed by Chaves et al. (2003). Under T3, soluble sugars and NSCs decreased to their lowest levels at R48h with no significant change in starch content (**Figure 5**), indicating that severe low temperatures blocked both glycolysis (inhibited hexokinase activity) and starch synthesis (inactivated ADP-glucose pyrophosphorylase) pathways (Peter, 2011), leading to collapse of the carbon metabolic system.

Dynamic responses of the antioxidant system showed clear stress gradient characteristics: after 24h of low-temperature stress, CAT, SOD, and POD activities, as well as MDA content, increased significantly with decreasing temperature, reaching maximum values under T3 (**Figure 3**), indicating that *O. lagopus* constructs a multi-level ROS scavenging network by upregulating antioxidant enzyme activities (Liu et al., 2025a). This synergizes with the 50.32% increase in Fo under T3 (**Figure 2A**) — a phenomenon of PSII reaction center closure—to jointly reduce superoxide anion (O_2_·^−^) production. MDA accumulation (**Figure 3**) reflects that 3°C low temperatures induce peroxidation of unsaturated fatty acids in thylakoid membranes, disrupting membrane integrity and forming a causal chain with the continuous decrease in chlorophyll a: membrane damage leads to disintegration of chlorophyll-protein complexes, exacerbating the collapse of the light-harvesting system (Calzadilla et al., 2025). Antioxidant strategies diverged significantly during recovery: under T7, both CAT and POD activities were significantly reduced during rewarming compared to L24h (**Figure 3**), suggesting that after mild low temperatures, plants reduce metabolic consumption by lowering antioxidant enzyme activities and reallocate resources to photosynthetic apparatus repair (e.g., LHCII complex reassembly). This coincides with the recovery of chlorophyll b, which reflects the trade-off between ‘oxidative defense and photosynthetic repair’ (Dong et al., 2001; Vighi et al., 2017). Under T5, CAT and POD activities continued to decrease, but MDA content only declined after R24h, indicating that *O. lagopus* adopts a balanced strategy of “enzyme activity downregulation - reduced membrane damage” under moderate low temperatures, maintaining membrane stability while avoiding overactivation of the antioxidant system—consistent with the “conservative repair” pattern characterized by persistently low Fv/Fm but stable chlorophyll content (Zhang et al., 2020). Under T3, MDA content increased by 20.67% at R24h while antioxidant enzyme activities continued to decrease, revealing that PSII electron transport recovered during rewarming but thylakoid membrane repair lagged, leading to ROS outbursts. Antioxidant enzymes lost activity due to protein denaturation (e.g., CAT subunit dissociation) (Hu et al., 2012).

Responses of osmotic regulatory substances further confirmed stress gradient differences: PRO accumulated significantly only under T3 (reaching 55.04 mg·g^−1^), exerting dual roles in osmotic regulation and hydroxyl radical (·OH) scavenging, forming a complementary defense with MDA surge (Lei et al., 2006). SP content increased in all low-temperature treatments, showing functional differentiation: under T7, SP may primarily consist of heat shock proteins (HSPs), which protect PSII by stabilizing D1 protein conformation (Wang et al., 2004; Vierling, 1991); under T3, SP may be associated with antifreeze protein (AFPs) synthesis, mitigating membrane damage by inhibiting ice crystal growth (Campbell and Close, 1997; Close, 1996). During recovery, T3 treatment maintained high PRO concentrations to cope with persistent osmotic stress, synergizing with MDA-rebound oxidative stress (Chaves et al., 2003); T5 treatment showed a rapid decrease in PRO, synchronous with MDA decline, suggesting resource prioritization for photosynthetic repair after osmotic stress alleviation; SP decrease under T7 may result from HSP degradation and recycling after completing repair functions, while SP decrease under T3 stems from insufficient synthetic substrates due to carbon metabolic collapse, exacerbating membrane damage.

In summary, the physiological and biochemical responses of *O. lagopus* seedlings exhibit a clear low-temperature threshold: 7°C stress enables full recovery through metabolic reconfiguration; 5°C induces “conservative repair” to maintain homeostasis; 3°C triggers coordinated collapse of carbon metabolism and the antioxidant system. This gradient response pattern reveals the “primitive strategy” of tropical tree species in low-temperature adaptation—relying on metabolic plasticity rather than active cold acclimation—providing key evidence for understanding plant stress resistance mechanisms under climatic zone differentiation.

### Primary adaptation strategies of *O. lagopus* seedlings to low-temperature stress and recovery time

Adaptation strategies of *O. lagopus* seedlings to low temperatures show significant temperature gradient differentiation. Principal component analysis (PCA) revealed a coordinated regulatory network involving “oxidative defense-carbon allocation - photosynthetic repair” (**Figure 6**). Under severe low temperatures (T3, 3°C), plants activate an “emergency defense” strategy: scavenging ROS through a surge in antioxidant enzyme activities (SOD, CAT), while maintaining basic osmotic pressure via soluble sugars and NSCs. This synergistic response mitigates damage through dual pathways: the antioxidant enzyme system dismutates O_2_·^−^ and decomposes hydrogen peroxide (H_2_O_2_) to reduce oxidative stress, while soluble sugars protect PSII reaction center structures by maintaining cell osmotic pressure (with Fv showing significant weight in PC1). However, this strategy only delays damage temporarily and cannot reverse photosynthetic system collapse caused by thylakoid membrane lipid peroxidation, which is closely related to the lack of cold acclimation-related genes (e.g., COR family) in tropical tree species, resulting in impaired repair capacity (Xiong et al., 2002; Ding et al., 2019). Under moderate low temperatures (T5, 5°C), a “conservative carbon allocation” strategy is adopted: starch accumulates with a decreased soluble sugar/starch ratio for energy storage, while increased carotenoids content enhances photoprotection (PC2 in PCA is dominated by carbon allocation indices and carotenoids, **Figure 6A**). Its homeostasis maintenance mechanism involves two aspects: at the carbon metabolic level, assimilates are prioritized for starch storage to reduce growth-related consumption, preserving energy for subsequent repair; at the photoprotective level, carotenoids enhance non-photochemical quenching (NPQ) through the xanthophyll cycle, dissipating excess light energy to avoid secondary damage to PSII (Demmig-Adams et al., 1996). This strategy differs from active cold acclimation in temperate plants and is more likely a passive metabolic adjustment of tropical tree species in response to occasional low temperatures.

Strategy shifts during the recovery phase exhibit time dependence: at R12h, PC1 is dominated by antioxidant enzymes (POD, SOD) and photosynthetic parameters (Fv, Fm) (**Figure 6B**), indicating plants prioritize downregulating oxidative defense intensity while initiating PSII reaction center repair (Fv/Fm recovery). Under T7, soluble sugars increased by 40.2% at R12h compared to L24h (**Figure 5A**), suggesting rapid decomposition of energy reserves into soluble sugars to provide carbon skeletons and energy for D1 protein synthesis—reflecting a resource allocation logic of “energy prioritization for repair.” At R24h, core indices of PC1 are POD, SOD, Fv/Fo, and chlorophyll a (**Figure 6C**), corresponding to chlorophyll b recovery to control levels and a 20.3% increase in Fv/Fo under T7, indicating synchronous improvement in PSII light energy transfer efficiency and reaction center activity. NSCs, soluble sugars, and the soluble sugar/starch ratio become core indices in PC2, consistent with the significant increase in this ratio under T5, reflecting a shift in carbon allocation from “starch storage” to “soluble sugar energy supply” to provide sustained energy for LHCII complex reassembly. At R48h, PC1 includes POD, MDA, Fv, and chlorophyll a (**Figure 6D**), revealing residual damage under T3: a 20.67% rebound increase in MDA content and continued decrease in chlorophyll a indicate accumulated thylakoid membrane lipid peroxidation products still inhibit the photosynthetic system. PC2 is dominated by PRO, NSCs, and soluble sugars, corresponding to PRO maintenance at 58.25 mg·g^−1^ and stable low-level NSCs under T3, indicating plants maintain basic cell functions through proline-mediated osmotic regulation and limited energy reserves, entering a “stress tolerance” state.

From the temperature gradient perspective, T7 exhibits “low damage - rapid repair” characteristics: only mild activation of SOD and POD during stress, with limited oxidative damage; rapid recovery of chlorophyll b after rewarming and a surge in soluble sugars at R24h, with PCA showing synchronous increases in PC1 (photosynthetic repair) and PC2 (carbon metabolism) scores. This strategy achieves efficient recovery through precise resource allocation, reflecting adaptive optimization to mild occasional low temperatures. T5 maintains “conservative repair”: PRO content decreases rapidly after rewarming, with stable soluble sugar/starch ratio; PCA shows the lowest dispersion of indices at R48h, reflecting retention of repair potential by reducing metabolic consumption—consistent with the “static response” ecological strategy of tropical tree species (Aragón et al., 2023). T3 falls into a “damage-dominated - repair-failed” state: MDA content rebounds and chlorophyll a continues to decrease after rewarming, confirming the inevitability of irreversible damage.

## Conclusion

*O. lagopus* seedlings form a hierarchical adaptation strategy to low-temperature stress and recovery through the integrated and synergistic responses of the photosynthetic system, antioxidant defense, osmotic regulation, and carbon metabolism. The response of *O. lagopus* to low-temperature stress exhibits a temperature threshold effect: 7°C represents a “reversible repair threshold,” where complete recovery of the photosynthetic system is achieved through rapid chlorophyll synthesis and downregulation of antioxidant enzymes. 5°C acts as a “metabolic homeostasis threshold,” maintaining energy balance through starch storage and carotenoids-mediated photoprotection. 3°C constitutes a “damage collapse threshold,” triggering irreversible damage due to ROS outbursts and carbon source depletion.

The recovery phase shows time-dependent strategic shifts: In the early stage (12 h), NSCs are prioritized for decomposition into soluble sugars to supply energy for D1 protein synthesis in PSII. In the middle stage (24 h), synchronous repair of photosynthetic pigments and light energy transfer efficiency occurs. In the late stage (48 h), under 3°C treatment, seedlings enter a “stress tolerance” state, maintaining survival solely through proline and residual energy reserves.

In summary, the adaptation mechanism of *O. lagopus* is an outcome of long-term adaptation of tropical tree species to their native habitats. In environments with high annual average temperatures and occasional low temperatures, it has evolved a three-level strategy: “efficient repair under mild low temperatures, homeostasis maintenance under moderate low temperatures, and stress tolerance under severe low temperatures.” This strategy avoids the high energy costs associated with cold acclimation in temperate tree species while enabling responses to short-term environmental fluctuations. These findings not only provide empirical evidence for understanding the multi-system synergistic mechanisms of stress resistance in tropical plants but also offer a theoretical basis for the conservation and management of tropical vegetation under climate change.

## Data Availability

The original contributions presented in the study are included in the article/supplementary material. Further inquiries can be directed to the corresponding author.
